# Dispersions of Goethite Nanorods in Aprotic Polar Solvents

**DOI:** 10.3390/ma10101191

**Published:** 2017-10-17

**Authors:** Delphine Coursault, Ivan Dozov, Christophe Blanc, Maurizio Nobili, Laurent Dupont, Corinne Chanéac, Patrick Davidson

**Affiliations:** 1 Laboratoire Charles Coulomb, CNRS, Université de Montpellier, 34095 Montpellier, France; dcoursault@uchicago.edu (D.C.); i.dozov.vre@free.fr (I.D.); christophe.blanc@umontpellier.fr (C.B.); maurizio.nobili@univ-montp2.fr (M.N.); 2 Laboratoire de Physique des Solides, CNRS, Université Paris-Sud, Université Paris-Saclay, 91405 Orsay CEDEX, France; 3 IMT Atlantique, Optics Department, Technopôle Brest-Iroise, CS 83818, 29238 Brest CEDEX 3, France; laurent.dupont@telecom-bretagne.eu; 4 Sorbonne Universités, UPMC Univ. Paris 06, CNRS, Collège de France, Laboratoire de Chimie de la Matière Condensée de Paris, 4 place Jussieu, 75005 Paris, France; corinne.chaneac@upmc.fr

**Keywords:** liquid crystals, colloids, nanoparticles, electro-optics, X-ray scattering, Kerr effect

## Abstract

Colloidal suspensions of anisotropic nanoparticles can spontaneously self-organize in liquid-crystalline phases beyond some concentration threshold. These phases often respond to electric and magnetic fields. At lower concentrations, usual isotropic liquids are observed but they can display very strong Kerr and Cotton-Mouton effects (i.e., field-induced particle orientation). For many examples of these colloidal suspensions, the solvent is water, which hinders most electro-optic applications. Here, for goethite (α-FeOOH) nanorod dispersions, we show that water can be replaced by polar aprotic solvents, such as *N*-methyl-2-pyrrolidone (NMP) and dimethylsulfoxide (DMSO), without loss of colloidal stability. By polarized-light microscopy, small-angle X-ray scattering and electro-optic measurements, we found that the nematic phase, with its field-response properties, is retained. Moreover, a strong Kerr effect was also observed with isotropic goethite suspensions in these polar aprotic solvents. Furthermore, we found no significant difference in the behavior of both the nematic and isotropic phases between the aqueous and non-aqueous dispersions. Our work shows that goethite nanorod suspensions in polar aprotic solvents, suitable for electro-optic applications, can easily be produced and that they keep all their outstanding properties. It also suggests that this solvent replacement method could be extended to the aqueous colloidal suspensions of other kinds of charged anisotropic nanoparticles.

## 1. Introduction

Colloidal liquid crystals are suspensions of anisotropic nanoparticles, such as nanorods or nanoplates, which in a given range of concentration spontaneously organize into liquid-crystalline phases. The latter are usually classified into the nematic phases with long-range orientational order and the lamellar and columnar phases that also have long-range positional order in one or two dimensions, respectively. On the one hand, colloidal liquid crystals, like suspensions of filamentous viruses, are interesting systems to test statistical mechanics models of liquid-crystalline ordering [1,2]. On the other hand, they can be used to produce functional materials for industrial applications such as polymer fibers with remarkable mechanical properties [3]. The so-called “mineral liquid crystals”, obtained by dispersing mineral anisotropic nanoparticles in a solvent, combine the usual properties (fluidity and anisotropy) of liquid crystals with electronic properties more commonly found in mineral compounds, such as magnetism, electric conductivity or luminescence [1,4,5,6]. For example, aqueous suspensions of goethite nanorods have been widely studied in recent decades because of their peculiar magnetic properties and their very rich mesomorphism that includes, in addition to the common nematic, lamellar, and columnar phases, the quite elusive biaxial nematic phase [7,8,9,10,11,12,13].

At somewhat lower concentrations, the usual isotropic phase, where the nanoparticles have no spontaneous orientational order, often also shows interesting properties. Goethite suspensions can again be used to illustrate this point as they show very strong birefringence when they are submitted to a magnetic field (Cotton-Mouton effect) or an electric field (Kerr effect) [14,15]. These effects arise from the orientational ordering of the goethite nanorods induced by either the magnetic or the electric field [8,9]. In spite of the large particle dimensions compared to molecular liquid-crystals, because the colloidal suspensions are dilute, cooperative effects are less important than in liquid-crystalline phases at higher concentrations, resulting in faster response times, on the order of tens of milliseconds. The large Kerr effect of these isotropic colloidal suspensions could therefore be used for electro-optic applications that do not require very fast response times, such as spatial modulators and tunable filters used in the telecommunication industry [16,17]. A major advantage of this approach is that there is no need of controlling the texture and anchoring of the liquid crystal, which is one of the main issues involved in the design of liquid-crystal-based electro-optic devices. Also, after scale-up, the production of mineral nanoparticles like goethite nanorods is likely to become much cheaper than that of the mixtures of liquid-crystalline molecules currently used in the industry.

However, one serious drawback that hinders the application of aqueous dispersions of mineral nanoparticles is that water has a strong absorption band in the infrared region just in the wavelength range (1300–1550 nm) used by the telecommunication industry. Water therefore needs to be replaced by another solvent where the particles would still be stable from the colloidal point of view. The colloidal stability of goethite aqueous dispersions is ensured by long-range electrostatic repulsions between the nanorods due to their positive electrical surface charge at acidic pH (~pH = 3), far away from the point of zero charge (~pH = 9). Moreover, at the microscopic scale, one of the most efficient mechanisms of the Kerr effect arises from the polarization of the counter-ion clouds around the particles. Therefore, water must be replaced by a polar solvent of large dielectric constant. Moreover, because a large ionic conductivity leads to highly detrimental local heating, we decided to turn to aprotic organic solvents.

In this article, we report on our investigation of the use of different aprotic polar solvents to produce stable colloidal suspensions of goethite nanorods that still display the interesting features of their aqueous counterparts, namely: (i) the presence of a nematic phase; (ii) its alignment in an electric field; and most importantly (iii) a strong Kerr effect in the isotropic phase.

## 2. Results and Discussion

### 2.1. Colloidal Stability of Goethite Nanorod Suspensions

**Figure 1:**
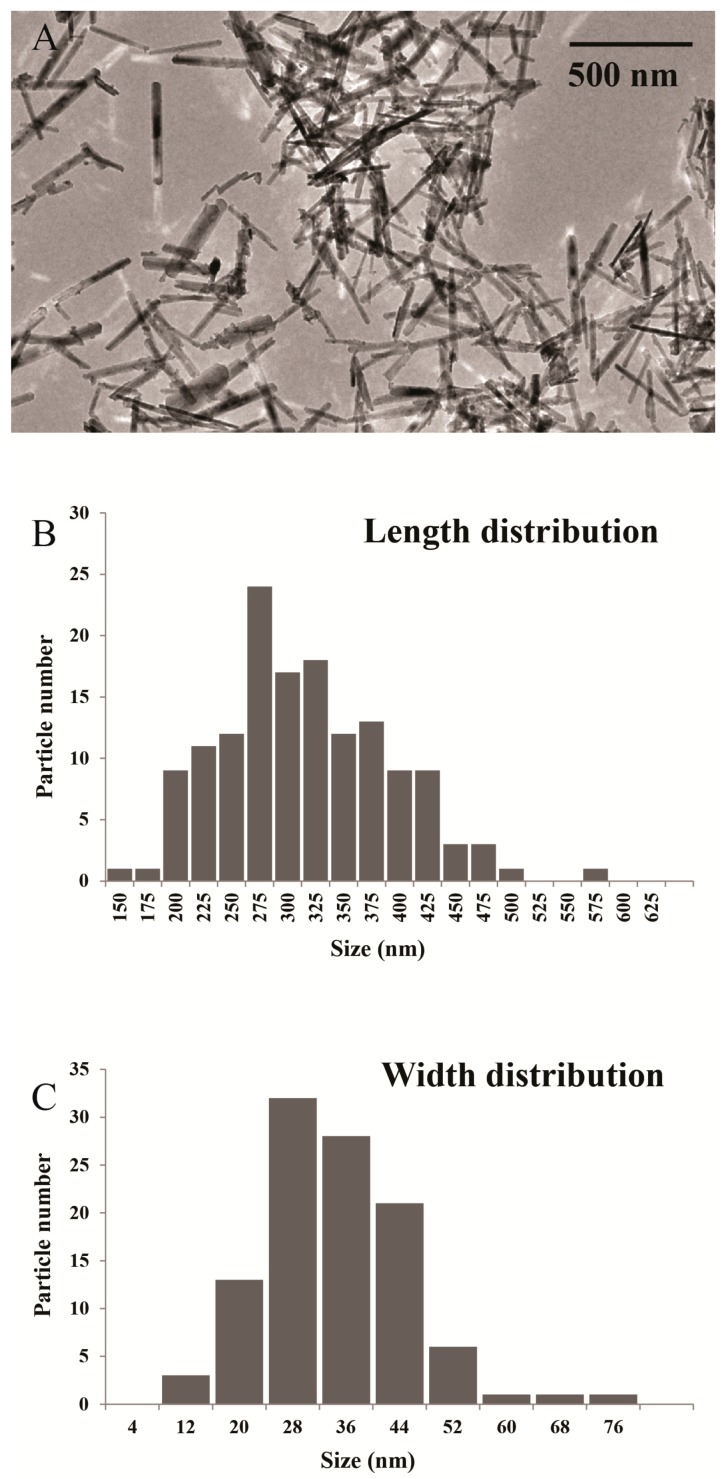
(**A**) Transmission electron microscopy (TEM) image of goethite nanorods; (**B**) Histogram of nanorod length distribution; (**C**) Histogram of nanorod width distribution.

Goethite nanorods were synthesized by following a well-described procedure [18,19] and were imaged by transmission electron microscopy (Figure 1). Their average length is 302 nm with a standard deviation of 75 nm while their mean diameter is 30 nm with a standard deviation of 11 nm.

The nanorods were dispersed in different polar solvents at various volume fractions, φ. The dispersions were poured in glass vials and were left undisturbed for several days to assess visually their colloidal stability. The following solvents were considered and compared: H_2_O, D_2_O (heavy water), dimethylsulfoxide (DMSO), *N*-methyl-2-pyrrolidone (NMP), dimethylformamide (DMF), propylene carbonate (PC), and acetonitrile (AN). Table 1 summarizes some of their physical properties. D_2_O was considered because its light absorption spectrum does not show a band in the telecom window thanks to the mass difference between hydrogen and deuterium atoms. Apart from H_2_O and D_2_O that lead to stable colloidal suspensions, we observed that the suspensions of goethite nanorods were only stable in DMSO, NMP, and PC. Suspensions in AN immediately showed particle aggregation, whereas particles suspended in DMF aggregated more slowly and lacked long-term colloidal stability, even though they could be redispersed by ultrasonic treatment. Nanoparticle aggregation shows up by the formation of a thin layer of dense goethite brown sediment in coexistence with a large clear supernatant on top of it. To date, from a physical chemistry perspective, we do not understand why goethite suspensions are not stable in AN and DMF.

Goethite nanorod dispersions in PC were not investigated in great detail in spite of their good electro-optic properties, like stable optical response and absence of degradation in high fields, because they showed response times larger than 100 ms, even at the low volume fraction of 0.3%, which is too long for applications. Therefore, in the following, we only systematically compare suspensions in H_2_O, D_2_O, DMSO, and NMP.

### 2.2. Existence of a Nematic Phase

**Figure 2:**
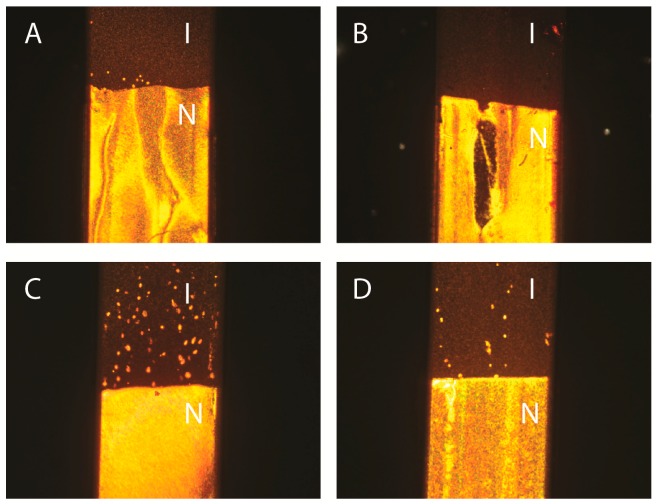
Photographs in polarized-light microscopy of biphasic isotropic/nematic (I/N) suspensions in flat glass capillaries. (**A**) H_2_O, φ = 3.2%; (**B**) D_2_O, φ = 5.5%; (**C**) DMSO, φ = 2.7%; (**D**) NMP, φ = 4.8%. (The polarizer and analyzer directions are parallel to the sides of the photographs; the capillaries are 1 mm wide.)

We first checked that the nematic phase still appears in these other solvents at volume fractions comparable to that observed in H_2_O. Figure 2A–D shows photographs in polarized light microscopy of samples in flat glass capillaries, stored vertically. All these samples look similar as they display the expected isotropic/nematic (I/N) phase coexistence which is the hallmark of the first-order I/N phase transition. This phase transition, in polar solvents, is classically explained by statistical mechanics models that include electrostatic interactions between particles. This theoretical approach does not rely on any specific assumptions about the precise molecular nature of the solvent which is only considered through its physical properties, such as its dielectric constant. Therefore, the appearance of the nematic phase not only in water but also in other polar solvents is not surprising. Note that nematic “tactoids” (i.e., nematic droplets in the isotropic phase) are visible on some of the photographs. Such observations mean that all samples reach thermodynamic equilibrium and that gelation, which is a usual problem met with polar solvents, only occurred at higher volume fractions. Moreover, close observation of both phases reveals the presence of strong Brownian motion in the isotropic phase and strong director fluctuations in the nematic phase, which confirms that the samples do not gel. (This is not necessarily the case with all solvents since, for example, goethite suspensions in ethylene glycol did not display a well-developed nematic phase, due to gelation.) For all four solvents examined here, a common planar (degenerate) texture is observed in the nematic phase, as expected, for entropic reasons, for nematic phases of nanorods confined between two glass plates [20]. (The same observations were made both for flat optical cells of a few microns thickness and for optical capillaries of thickness 50–100 microns.)

Samples in flat glass capillaries (50 μm thick, 1 mm wide, and ~80 mm long) were stored in vertical position, in the field of gravity. Consequently, all these samples displayed a stable vertical concentration gradient, which makes the exact volume fraction at the transition difficult to determine. (We chose to prepare samples of roughly the same overall volume fractions for the four goethite/solvent systems because, in spite of the density gradient, we expected the I/N transition to occur at similar concentrations for all of them, which is indeed the case.) This gradient is not the sign of any particle aggregation but it is due to the competition between particle sedimentation and Brownian motion. In first approximation, the concentration profile may be considered exponential with a typical length scale l_g_ = k_B_T/V∆ρ, where k_B_ is the Boltzmann constant, V the particle volume and ∆ρ the mass density contrast between the particle and the solvent. For goethite nanoparticles and all solvents considered here, l_g_ ~ 2 mm, which leads to a large concentration variation along the glass capillary. Such phenomenon was already exploited to quickly map out the phase diagram of goethite suspensions in water [11]. This simple reasoning however neglects electrostatic interactions and many-particle effects and, in practice, the concentration profile is not quite as steep. Nevertheless, in order to minimize concentration variations among samples in different solvents and make comparisons as meaningful as possible, we always made all measurements close to the isotropic/nematic phase boundary, in both phases.

**Figure 3:**
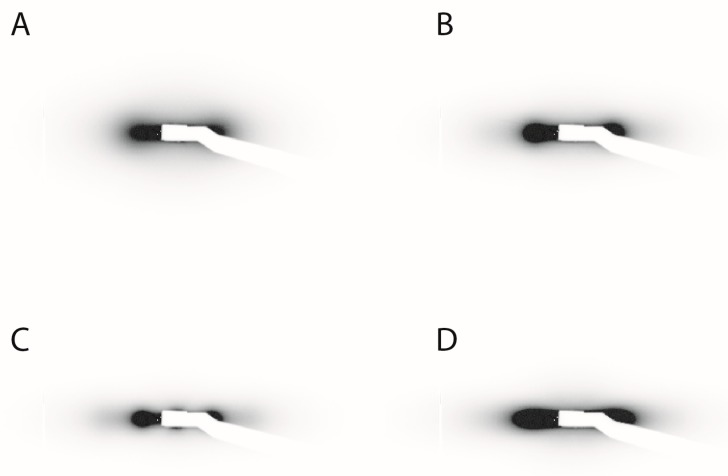
SAXS patterns of the nematic phase aligned by an a.c. electric field for all samples shown in Figure 2. (**A**) H_2_O, φ = 3.2%; (**B**) D_2_O, φ = 5.5%; (**C**) DMSO, φ = 2.7%; (**D**) NMP, φ = 4.8%. (The electric field was applied along the vertical direction.)

**Figure 4:**
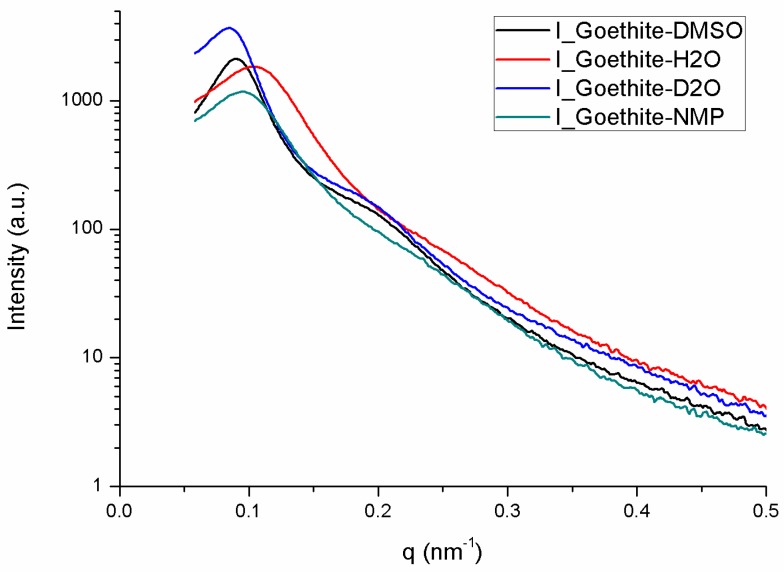
Curves of scattered intensity versus scattering vector modulus derived from the SAXS patterns of Figure 3.

Small-angle X-ray scattering (SAXS) experiments were also done to prove the nematic nature of the liquid-crystalline phase detected by polarized-light microscopy. Because the SAXS patterns of an isotropic phase and an unaligned nematic phase are too similar to be easily distinguished, the nematic phase had to be aligned by applying an electric field in-situ on the SAXS beamline. The scattering patterns of the nematic parts of the same four biphasic samples shown in Figure 2 are all strikingly alike (Figure 3A–D). As expected, these patterns only display diffuse scattering spots, which shows that these phases lack any long-range positional order of the goethite nanorods. Moreover, the patterns are highly anisotropic, which is due to a strong orientational order of the nanorods. Hence, the liquid-crystalline phase is indeed nematic, whatever the precise nature of the solvent. The diffuse scattering spots arise from the liquid-like, short-range, positional correlations of the nanorods in the plane perpendicular to the director. Their location shows that, in all solvents, the nanorods align parallel to the a.c. electric field. Moreover, the width of the spots, along an azimuthal-angle profile of scattered intensity, is related to the nematic scalar order parameter, S [21]. Such profiles are quite similar for all four solvents (data not shown), which shows that the strength of the spontaneous orientational order of the nanorods in the nematic phase is not much affected by the nature of the solvent. Indeed, using a classical method to derive S from the azimuthal profile [21], the following values of S are obtained: S = 0.92 ± 0.05 in H_2_O; S = 0.95 ± 0.05 in D_2_O; S = 0.93 ± 0.05 in DMSO; and S = 0.84 ± 0.05 in NMP. Such values of S are much larger than those found for the nematic phases of molecular liquid crystals but are more common for colloidal liquid crystals. The value found in NMP seems to be a little lower than for the other three solvents but the difference is still within the error bars. Finally, the radial profiles, I(q), of the scattered intensity along the diffuse spots are also similar for all solvents (Figure 4). They all display a broad but fairly intense peak, with a much less pronounced second-order peak. These scattering peaks are a little weaker in the case of NMP but, apart from that, the similarity of the radial profiles means that the positional short-range order of the particles is also little affected by the precise nature of the solvents considered in this study.

### 2.3. Magneto-Optic Response of the Goethite Suspensions

**Figure 5:**
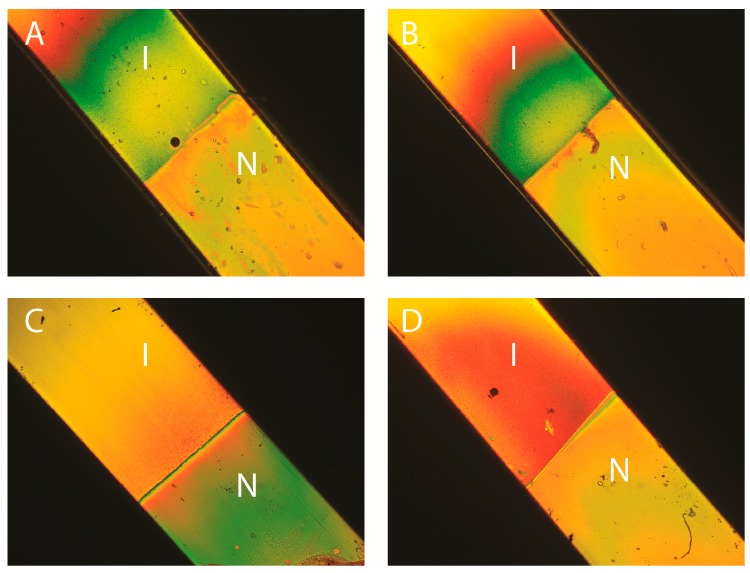
Photographs in polarized-light microscopy of the same biphasic (I/N) samples shown in Figure 2, submitted to a B = 0.2 T magnetic field perpendicular to the capillary axis. (**A**) H_2_O, φ = 3.2%; (**B**) D_2_O, φ = 5.5%; (**C**) DMSO, φ = 2.7%; (**D**) NMP, φ = 4.8%. (The polarizer and analyzer directions are parallel to the sides of the photographs; the capillaries are 1 mm wide.)

Applying a small magnetic field, B = 0.2 T, has a clear effect on the biphasic suspensions of goethite nanorods previously shown in Figure 2 (Figure 5A–D). In less than a minute, all the textural defects that were visible in the nematic part of the samples vanished and nematic single domains were obtained. Indeed, the optical extinction of the samples is perfect when the capillary axis is parallel to the axes of the polarizer or the analyzer. The isotropic part (in zero-field) of each sample becomes strongly birefringent in the magnetic field (Cotton-Mouton effect). Moreover, no clear difference in behavior between the samples in H_2_O, D_2_O, DMSO, and NMP could be observed. Therefore, the interesting magnetic properties of the goethite suspensions are completely preserved upon solvent exchange. This is not so surprising because these properties are a direct consequence of the nanorod magnetic features (remanent magnetic moment and anisotropy of magnetic susceptibility) which are little affected by the precise nature of the solvent.

### 2.4. Electro-Optic Response of the Goethite Suspensions

**Figure 6:**
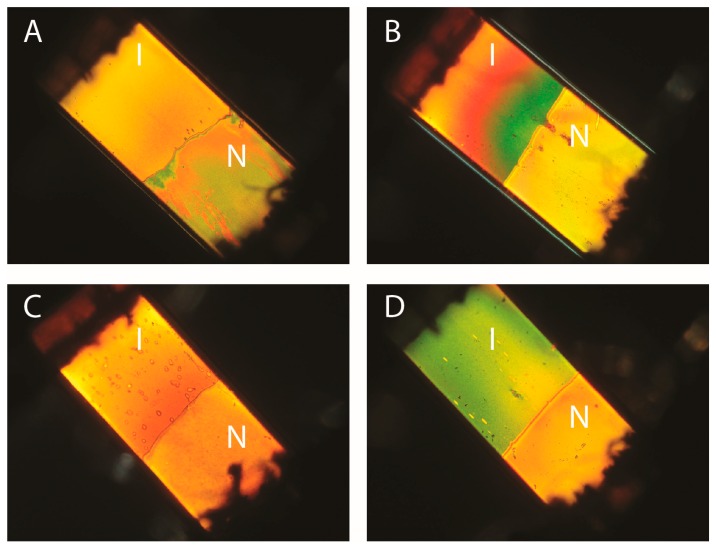
Photographs in polarized-light microscopy of the same biphasic (I/N) samples shown in Figure 2, submitted to an a.c. electric field along the capillary axis. (**A**) H_2_O, φ = 3.2%; (**B**) D_2_O, φ = 5.5%; (**C**) DMSO, φ = 2.7%; (**D**) NMP, φ = 4.8%. (The polarizer and analyzer directions are parallel to the sides of the photographs; the capillaries are 1 mm wide; the dark areas at the top and bottom of the capillaries are the electrodes used to apply the field.)

Figure 6A–D shows photographs of the same samples as in Figure 2 now submitted to an a.c. electric field. For all of them, the texture of the nematic phase has become planar and uniform in the field. The phase appears completely dark when the capillary axis is parallel to either the polarizer or analyzer directions whereas the phase appears brightest when the capillary axis lies at 45° from these directions. This confirms that the nematic phase is very well aligned by the electric field, as already inferred from the SAXS experiments described above. Moreover, for all four samples, the isotropic phase also appears very bright in the same geometry. This is precisely due to the Kerr effect that we wish to exploit for electro-optic applications. Qualitatively, we note that the Kerr effect is observed with the same intensity for all four samples.

**Figure 7:**
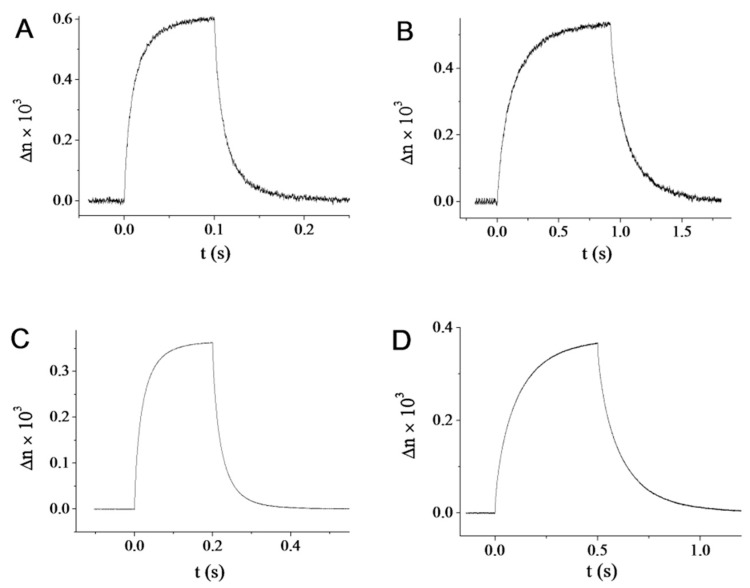
Field-induced birefringence in the isotropic phase for all samples shown in Figure 2. (**A**) H_2_O, φ = 3.2%, E = 13.8 V/mm, *f* = 700 kHz; (**B**) D_2_O, φ = 5.5%, E = 10.0 V/mm, *f* = 700 kHz; (**C**) DMSO, φ = 2.7%, E = 35.0 V/mm, *f* = 1 MHz; (**D**) NMP, φ = 4.8%, E = 35.0 V/mm, *f* = 1 MHz.

To study this effect more quantitatively, we made time-resolved measurements of the field-induced birefringence of samples submitted to bursts of high-frequency a.c. electric fields (Figure 7A–D). There again, all samples behave in a similar way. We studied the influence of both the field frequency and amplitude. Below some cut-off frequency, *f_c_*, the birefringence signal strongly decreases, which is due to the screening of the field by the mobile ions in the suspension. *f_c_* is in fact related to the conductivity of the suspension by the relation *f_c_* = *K_e_*/2πε_0_ε*_e_* where *K_e_* is the suspension conductivity, and ε_0_ and ε*_e_* are respectively the vacuum permittivity and the dielectric constant of the suspension. Therefore, using a suspension of lower conductivity allows working at lower frequency, an important feature in an applied perspective. We note that, as expected, the two aprotic solvents, DMSO and NMP, indeed show much lower *f_c_* than H_2_O and D_2_O (Table 2). Both the rise and decay of the field-induced birefringence can be fitted with exponential dependences. Such fits provide the rise time τ*_on_* and decay time τ*_off_* which are shown in Table 2. Upon increasing field amplitude, the birefringence first increases linearly with squared amplitude (strictly speaking, this is the Kerr effect). Then, the birefringence deviates from this dependence until it eventually saturates at high fields when all nanorods are aligned along the field direction.

Table 2 shows that the saturation field remains of the same order of magnitude for all samples. Similarly, both the saturated birefringence and specific birefringence do not seem to depend much on the solvent nature. The Kerr constants however seem to be a bit lower for the aprotic solvents than for H_2_O and D_2_O but they can also vary with the precise value of the local volume fraction where the measurement was made. In contrast, the value of the saturating field is independent of the local volume fraction and is inversely proportional to the excess polarizability of the particles in the solvent. We note that it is two–three times larger for NMP than for the other solvents even though it remains quite acceptable for applications. This larger value of the saturating field is most probably related to the lower value of ε*_r_* for NMP (Table 1) compared to those of DMSO, H_2_O, and D_2_O.

Our studies demonstrate that transferring goethite nanorods to aprotic polar solvents, like NMP and DMSO, is possible without loss of colloidal stability and that the dispersions thus produced keep the physical properties required for the electro-optic applications that we have in mind. Preliminary electro-optic experiments show that suspensions in NMP are indeed quite promising in this respect. From a more fundamental point of view, the influence of solvent properties on the colloidal stability and phase diagram of dispersions of anisotropic nanoparticles is a topic that focuses increasing interest [22,23,24]. In addition to the macroscopic properties of the solvent, like its dielectric constant and refractive index, other more subtle and detailed features, such as the ionization constant of the goethite surface OH groups in the different solvents, probably also matter. Reaching a better understanding of this topic will most probably require a systematic comparison of the behaviour of dispersions in different solvents, as we tried to illustrate in this article.

## 3. Materials and Methods

### 3.1. Goethite Synthesis

Goethite nanorods were synthesized by dissolution-recrystallization process of ferrihydrite in aqueous solution. First, 400 mL of Fe(NO_3_)_3_ (0.1 M) were hydrolyzed using NaOH solution (2 M) at room temperature to obtain fresh brown ferrihydrite. When the pH of the suspension reaches the value of 11, the mixture was aged for 3 weeks at room temperature without stirring. After this delay, the ochre precipitate was separated from solution by centrifugation and washed three times with water. A stable colloidal suspension of non-aggregated goethite nanorods was then obtained by dispersing the solid in acidic water (HClO_4_, pH = 2).

### 3.2. Electron Microscopy

Transmission electron microscopy (TEM) was used to confirm the rod shape of goethite nanoparticles and to determine their size distribution. A drop of the 1000-fold diluted goethite suspension was deposited on an amorphous carbon-coated copper grid and dried using an infrared lamp. TEM images were collected with a TWIN 120, Tecnai Spirit G2, (FEI, Hillsboro, OR, USA) electron microscope at 120 kV (Figure 1).

### 3.3. Goethite Solvent Exchange

In order to obtain a dispersion of goethite in D_2_O, an aliquot of aqueous suspension of goethite nanoparticles was centrifuged 30 min at high speed (7000 g). The sediment was then washed with D_2_O, centrifuged again and finely dispersed in pure D_2_O. The dispersion state was improved by submitting the suspension for two minutes to the ultrasounds produced by an immersion probe operating in pulse mode. For the exchange with others solvents, such as dimethyl sulfoxide or *N*,*N*-dimethylformamide, the same procedure was applied, except that ethanol was used as the washing solvent. If necessary, more dilute samples were prepared by dilution with weighed amounts of pure solvent.

### 3.4. Optical Microscopy

Samples were filled by capillarity into flat glass capillaries (VitroCom, Mountain Lakes, NJ, USA) of 50 μm thickness and 1 mm width. They were examined by polarized light microscopy using an optical microscope (Olympus BX51, Tokyo, Japan) and their textures were photographed using an Olympus digital camera (Olympus, Hamburg, Germany).

### 3.5. Electro-Optics

Transient birefringence measurement is a classical method [15,25,26] to study the electric field-induced order in colloidal suspensions. However, instead of the classical Kerr-cell, we use a specially developed setup [27] allowing us to work with very small samples (about 10 mm^3^) and to apply the field without direct contact of the electrodes with the suspension. In this way, we avoid the electrolysis and the electrochemical degradation issues typical for the electro-optic studies in aqueous (or other polar) solvents. In addition, because of the weak currents in the sample, this technique allows us to apply moderately strong fields (~1 V/μm) using very simple and standard instrumentation.

The colloidal dispersion is contained in a flame-sealed glass flat capillary with 0.05 × 1 mm^2^ cross-section in the present case. The electric field is applied along the capillary axis by a pair of external electrodes, typically separated by an inter-electrode distance, *L_e_* = 2 mm. In practice, the electrodes are made of a thin aluminum foil, which is gently pressed on the external glass wall of the capillary by a foam cushion, mounted in a home-made sample-holder. High-frequency (up to *f* = 1 MHz) sinusoidal voltage bursts are applied to the electrodes. The electric signal is generated by an arbitrary waveform generator (TTi TGA1241, Fort Worth, TX, USA) which controls the burst duration, frequency and repetition rate. This signal is amplified up to amplitude *U*_0_ = 400 V by a wide-band amplifier (Krohn-Hite 7602M, Brockton, MA, USA). If necessary, the voltage is further amplified up to about 2000 V by a customized small-power transformer adapted to the frequency of the signal.

During the experiment, the sample-holder is placed on the stage of a polarizing microscope (Leitz Ortholux, Wetzlar, Germany) housing all the optical elements of the setup, which is described in detail elsewhere [27,28]. Briefly, we use an optical configuration with crossed polarizers and additional λ/4 phase-shift introduced by a Berek compensator (Olympus, Tokyo, Japan). Thus, the transmitted light intensity varies linearly with the phase-shift δ(E) induced by the electric field in the suspension. The transmitted light is detected by a photomultiplier tube (PMT), which is also mounted on the microscope. The PMT signal is further amplified (with optional filtering of the high-frequency noise) using a wide-band differential amplifier AM 502, Tektronix, Beaverton, OR, USA. Finally, the signal is accumulated and averaged (up to 64,000 bursts) on a digital oscilloscope (Agilent DSO-X 2004A, Santa Clara, CA, USA). This setup allows us to measure the phase-shift δ(E) with a resolution better than 0.01 nm and its variation in time (with time-resolution better than 1 μs).

Due to the external electrodes used in our setup, the RMS field in the sample is not simply $U_{0}/(\sqrt{2}L_{e})$, as in the Kerr-cell setup, but is additionally attenuated, $E_{RMS}=C_{d}C_{s}(f)U_{0}/(\sqrt{2}L_{e})$ [27]. Here, *C_d_* is a correction factor, taking into account the dielectric constants of the glass wall and the solvent, as well as the geometry of the cell. The numerical calculation for the present experiments gives the typical value *C_d_* = 0.90, which is taken into account in the interpretation of the results. The frequency-dependent attenuation coefficient *C_s_(f)* accounts for the screening of the field by the conductivity charges accumulated on the inner side of the capillary wall in the vicinity of the electrodes. At low frequencies, *f* << *f_c_*, where $f_{c}=K_{s}/(2\pi \varepsilon_{0}\varepsilon_{s})$ is the relaxation frequency of the charges in the solvent with conductivity *K_s_* and dielectric constant ε*_s_*, the field is strongly attenuated, *C_s_(f)* ≈ 0. With increasing frequency, the field penetrates the sample better, as the conductivity charges cannot follow the sign-inversion of the field, and at *f* >> *f_c_*, the field penetrates completely in the solvent, without screening losses, *C_s_(f)* ≈ 1. Although *C_s_(f)* can also be calculated numerically [29], we prefer a more direct approach: we measure experimentally, as a function of the frequency, the voltage *U*_0_ required to obtain a constant transient birefringence, corresponding to the same E_RMS_ in the sample; then the *C_s_(f)* curve is calculated taking into account that *C_s_(f)* ≈ 1 on the high-frequency plateau of the curve [27]. When the frequencies *f* >> *f_c_* are accessible with our setup, we measure the induced birefringence at these frequencies, neglecting the screening losses. Otherwise, we use the optimal frequency at which the highest $C_{s}(f)U_{0}$ value is obtained experimentally and then we correct the internal field value using the measured *C_s_(f)* function.

### 3.6. Synchrotron Small-Angle X-Ray Scattering

SAXS experiments were performed at the Swing beamline of the SOLEIL synchrotron radiation facility (Saint-Aubin, France). Measurements were made using a fixed energy of 12.0 keV and a sample-to-detector distance of 6.56 m. The typical accessible range of scattering vector modulus q was 10^−2^–1 nm^−1^ (q = (4π/λ)sin θ, where 2θ is the scattering angle and λ = 0.1033 nm is the wavelength). Scattering patterns were recorded on a CCD camera (AVIEX 170170, Naperville, IL, USA) formed by four detectors and placed in a vacuum detection tunnel. The same sample cell that was used for the electro-optic measurements was fitted on the beamline with the flat faces of the capillaries set perpendicular to the X-ray beam, to record the SAXS patterns of samples submitted to the a.c. electric field. The SAXS patterns were acquired, reduced, and normalized using standard beamline procedures.

## Figures and Tables

**Table 1:** Abbreviations (Abb.) and physical properties of the solvents used in this study. *n*: refractive index; ε_r_: dielectric constant; T_m_: melting temperature; T_b_: boiling temperature.

Solvents	Abb.	*n*	ε_r_	T_m_ (°C)	T_b_ (°C)	Viscosity (mPa·s)	Surface Tension (dynes/cm)
Dimethylsulfoxide	DMSO	1.479	48	19	189	2.0	42.9
*N*-methyl-2-pyrrolidone	NMP	1.470	32	−24	204	1.67	44.6
Dimethylformamide	DMF	1.430	37	−60	152	0.79	34.4
Propylene Carbonate	PC	1.421	64	−49	242	2.50	40.9
Acetonitrile	AN	1.344	37.5	−44	82	0.37	28.7

**Table 2:** Electro-optic properties of isotropic goethite suspensions in different solvents. φ: volume fraction; ∆n_sat_: saturated birefringence (∆n_sat_ = ∆n(E→∞)); ∆n_sp_: specific birefringence (∆n_sp_ = ∆n_sat_/φ); E_sat_: saturating field E_sat_ = (∆n_sat_/C_K_)^1/2^; C_K_ = ∆n(E)/E^2^|_E→0_ is one of the definitions of the Kerr coefficient; *f_c_* is the relaxation frequency of the conductivity charges in the solvent; τ*_on_* and τ*_off_* are respectively the rise and decay times of the electro-optic signals.

Solvent	φ (%)	∆n_sat_	∆n_sp_	E_sat_ (V/mm)	C_K_ (m^2^/V^2^)	*f_c_* (kHz)	τ*_on_* (ms)	τ*_off_* (ms)
H_2_O	3.2	0.031	0.97	88	4.0 × 10^−12^	140	13.6	13.1
D_2_O	5.5	0.032	0.58	60	7.3 × 10^−12^	260	130	130
DMSO	2.7	0.023	0.99	118	1.2 × 10^−12^	20	26	27
NMP	4.8	0.0154	0.32	186	3.1 × 10^−13^	20	102	101
